# Integrated Proteotranscriptomics of the Hypothalamus Reveals Altered Regulation Associated with the *FecB* Mutation in the *BMPR1B* Gene That Affects Prolificacy in Small Tail Han Sheep

**DOI:** 10.3390/biology12010072

**Published:** 2022-12-30

**Authors:** Xiangyu Wang, Xiaofei Guo, Xiaoyun He, Ran Di, Xiaosheng Zhang, Jinlong Zhang, Mingxing Chu

**Affiliations:** 1 Key Laboratory of Animal Genetics, Breeding and Reproduction, Ministry of Agriculture and Rural Affairs, Institute of Animal Science, Chinese Academy of Agricultural Sciences, Beijing 100193, China; 2 Institute of Animal Husbandry and Veterinary Medicine, Tianjin Academy of Agricultural Sciences, Tianjin 300381, China

**Keywords:** *FecB*, Small Tail Han sheep, fertility, hypothalamus, transcriptome, proteome, integration analysis

## Abstract

**Simple Summary:**

Increasing sheep litter size is essential to improve lamb production efficiency. In Small Tail Han sheep, the large variation in litter size is determined by segregation of the bone morphogenetic receptor type 1B (*BMPR1B*) gene. *BMPR1B* is the major gene for fertility that affects the ovulation rate, and a prolific allele is *FecB^B^,* also called the *FecB* mutation. The mechanism of how the hypothalamus regulates ovulation number increase in sheep with the *FecB* mutation is not clear. Using a systems biology workflow in DIABLO, we integrated hypothalamic transcriptomics and proteomics data to predict the main fertility-related biomarkers (gamma-aminobutyric acid type A receptor subunit alpha 1, gamma-aminobutyric acid type A receptor subunit beta 2 and FKBP prolyl isomerase 1A) in the hypothalamus that may regulate changes in the ovulation rate of *FecB* mutant sheep by participating in gonadotrophin-releasing hormone synthesis and secretion. These results may provide potential genetic markers for breeding multi-lambing meat sheep These results also provide new insight into the reproductive endocrine regulation of fecundity and potential genetic markers of breeding for prolificacy in meat sheep.

**Abstract:**

The litter size and ovulation rate are different among ewes of different *FecB* genotypes in Small Tail Han sheep. These variants in reproductive phenotypes may be regulated by hormones released by the hypothalamic–pituitary–ovarian axis. However, there have been few reports on the hypothalamus regarding regulating an increase in ovulation in sheep with *FecB* mutation at different estrous stages. Thus, we examined the abundance of hypothalamus tissue protein profiles of six *FecB* mutant homozygous (BB) and six wild-type (WW) ewes at the luteal and follicular phases. We determined this abundance by tandem mass tag-based quantitative analysis and parallel reaction monitoring methods. Furthermore, an integrated proteotranscriptomic analysis was performed by the Data Integration Analysis for Biomarker discovery using the latent variable approaches for Omics studies (DIABLO) framework to examine biological processes and pathway alterations by the *FecB* mutant. The abundance of 154 proteins was different between the two estrous stages. Growth hormone and prolactin were particularly enriched in the neuroactive ligand–receptor interactions, the prolactin signaling pathway, and the PI3K-Akt signaling pathway which are related to hypothalamic function and reproduction. We combined proteome and transcriptome data from different estrous stages and genotypes. There is a high correlation (Pearson correlation coefficient = 0.99) between the two datasets in the first two components. We applied the traditional single-omic multivariate approach to obtain differentially abundant proteins and differentially expressed genes. The major fertility related biomarkers were selected using the two approaches mentioned above. Several key pathways (GABAergic synapse, neuroactive ligand–receptor interaction, estrogen and MAPK signaling pathways) were enriched, which are central to gonadotrophin-releasing hormone (GnRH) secretion and reproduction. A higher level of gamma-aminobutyric acid type A receptor subunit alpha1 (GABRA1) and gamma-aminobutyric acid type A receptor subunit beta2 (GABRB2) expression was observed in BB ewes as compared to WW ewes. This finding suggested that a greater production of GnRH during follicular development in BB ewes may explain the higher mature follicle number in mutant ewes. FKBP prolyl isomerase 1A (FKBP1A), which was a major feature factor in the proteome selected by DIABLO, was an important switch for activating the transforming growth factor beta (TGFβ) pathway, and its expression was higher in the WW ewes than in the BB ewes. We suggest that BB sheep maintain TGFβ pathway activity by reducing FKBP1A protein abundance. This innovative data integration in the hypothalamus may provide fresh insight into the mechanisms by which the *FecB* mutation affects sheep fertility, while providing novel biomarkers related to reproductive endocrinology in sheep breeding.

## 1. Introduction

The Booroola fecundity (*FecB*) gene has a major effect on the litter size and ovulation in sheep, and it was first found in Booroola Merino sheep [1]. In 2001, the causal mutation affecting sheep ovulation was finely mapped on chromosome 6 to the bone morphogenetic receptor type 1 B (*BMPR1B*) gene, which has an A746G mutation in the coding region. This mutation cause a change in amino acid at position 249 from glutamine to arginine (Q249R) [2,3,4]. In recent years, gene-edited sheep with the *FecB* mutation (p.Q249R), have been constructed by clustered regularly interspaced short palindromic repeat (CRISPR)/CRISPR-associated 9 technology. This technology has shown that the *FecB* mutation can greatly increase sheep fecundity [5,6]. Our previous work has demonstrated the *FecB* mutation is associated with the litter size of Small Tail Han (STH) sheep [7,8]. The *FecB* mutation in STH sheep greatly increases the litter size and ovulation in mutant homozygotes [9].

In sheep, the hypothalamic–pituitary–gonadal (HPG) axis is essential for follicle growth, maturation and ovulation. The central regulator of the HPG axis is gonadotrophin-releasing hormone (GnRH). GnRH is released by the hypothalamus, and controls the synthesis and release of luteinising hormone (LH) and follicle stimulating hormone (FSH) in the pituitary. Prior to ovulation, hormones from the follicles regulate GnRH secretion in the hypothalamus, followed by altered FSH and LH release in the pituitary [10]. FSH is also involved in the screening of the dominant follicles, and eventually, a preovulatory LH surge causes ovulation [11]. The mean plasma FSH concentration is higher in mutant homozygous (BB) sheep than in the wild genotypes in Merino, Romney and STH ewes [9,12]. Using the hypothalamic pituitary disconnected ovariectomized sheep model, a study showed that Romney ewes and Booroola Merino ewes with *BB* genotype had higher peripheral blood FSH than wild genotype ewes, possibly due to the induction of GnRH [13]. BMPR1B is a potent receptor for various BMP factors [14]. BMPR1B regulates the differentiation of follicles by FSH and also controls progesterone feedback to the hypothalamus and pituitary gland regulating FSH and LH secretion [15]. The effect of the *FecB* mutation on BMPR1B activity in the ovary has not been determined. *BMPR1B* is expressed in multiple sheep tissues. Thus, the *FecB* mutations may function in multiple tissues [16].

In recent years, multi-omics research strategies, such as comparative proteomics and transcriptomics analysis, have broadened the understanding of the functions of the hypothalamus and interactions of signal transduction of the hypothalamus involved in reproduction [17,18,19,20]. However, this knowledge is incomplete, and the molecular mechanism of hypothalamic involvement in the regulation of the litter size by the *FecB* mutation is still not well known. Systems biology and innovative data integration can unravel complex biology with new insights [21].

In the present study, tandem mass tag (TMT)-quantitative proteomics coupled with the parallel reaction monitoring (PRM) method were used to identify changes in the abundance of protein in hypothalamus tissue collected from STH ewes with different *FecB* genotypes in the luteal and follicular phases of the estrous cycle. Furthermore, an integrated analysis between proteomic and transcriptomic data from our previous study was used to screen core biomakers, signaling pathways and biological processes in the hypothalamus that regulate ovulation by the *FecB* mutation.

## 2. Materials and Methods

### 2.1. Experimental Design and Sample Preparation

**Figure 1:**
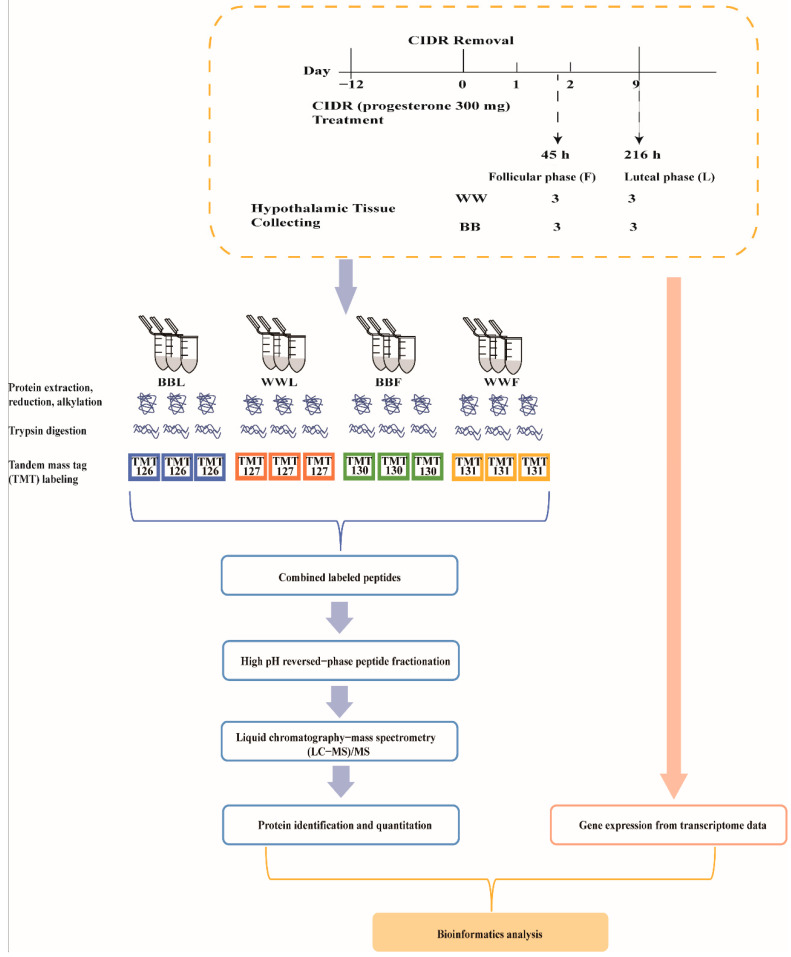
Sample grouping information overview of processing. Based on *FecB* genotypes, six STH ewes with the wild genotype (WW) and six *FecB* mutant homozygous (BB) were treated with controlled internal drug release (CIDR). After 12 days, the CIDR was removed from the ewes while the CIDR withdrawal time was recorded as 0 h. Hypothalamus tissue was collected from three WW ewes and three BB ewes euthanized at 45 h (follicular phase) (WWF, BBF). Subsequently, from three WW ewes and three BB ewes euthanized at 216 h (luteal phase) (WWL, BBL). The TMT quantitative proteomics analysis was employed to identify changes in the abundance of protein in hypothalamus tissues. Finally, bioinformatics analysis was performed in combination with downloaded transcriptomic data from the same samples.

Based on *FecB* genotypes, six ewes with wild (WW) genotype and six ewes with the BB genotype were selected from the STH core herd in Yuncheng County, Shandong Province using the TaqMan MGB assays [9]. These selected ewes were approximately 2–4 years old, of similar weight and not pregnant. They were fed in ad libitum and housed at a natural temperature and lighting. These 12 selected ewes were placed on controlled internal drug release (CIDR) (progesterone 300 mg) (InterAg Co., Ltd, Hamilton, New Zealand) for spontaneous estrous, and injected with vitamins A and D to protect the endometrium. After 12 days, the CIDR was removed from the ewes while the CIDR withdrawal time was recorded as 0 h. Then, three WW ewes and three BB ewes were euthanized at 45 h (follicular phase, F). Subsequently, three WW ewes and three BB ewes were euthanized at 216 h (luteal phase, L). Hypothalamus tissues were dissected immediately after euthanasia of ewes, and 12 tissues were snap frozen in liquid nitrogen and then transferred to −80 °C for storage until further experiments (Figure 1).

The hypothalamus tissues proteins were extracted using the SDT lysis method [22], and the bicinchoninic acid (BCA) protein assay (Pierce™ BCA Protein Assay Kit, Thermo Fisher Scientific, Waltham, MA, USA) was used to quantify protein concentrations [23]. Approximately 200 g of protein per sample was digested by trypsin with the use of a filter aided sample preparation method [24]. The peptide content was quantitated by ultraviolet spectrophotometry (optical density: 280) for the further proteomic experiments.

### 2.2. TMT Labeling and Peptide Fractionation Utilizing the High pH Reversed-Phase Approach

We labeled peptides (100 μg per sample) using the six plex tandem mass tag of the TMT Mass Tagging kit (Thermo Fisher Scientific, USA) according to the kit instructions. WW ewes at the luteal phase (WWL), BB ewes at the luteal phase (BBL), BB ewes at the follicular phase (BBF) and WW ewes at the follicular phase (WWF) were labeled with TMT-126, TMT-127, TMT-130 and TMT-131, respectively. Each group had three biological repeats (Figure 1).

The labeled peptides of 12 samples were mixed in an equal volume and fractionated into 10 fractions using the Pierce high pH reversed-phase fractionation kit (Thermo Fisher Scientific, USA). Briefly, 100 μg lyophilized mixed peptides were diluted to 300 L with 0.1% trifluoroacetic acid. The column was equilibrated using 0.1% trifluoroacetic acid and acetonitrile in water buffer, and diluted peptide solution was transferred to the column. We added 300 L of pure water for desalting and centrifuged the solution with a low speed. Subsequently, 10 peptide fractions were eluted by an increasing acetonitrile step-gradient elution. After vacuum drying, the eluted samples were re-solubilized with 0.1% formic acid, and the concentration was determined by measuring the absorbance at an optical density of 280.

### 2.3. Liquid Chromatography–Mass Spectrometry and Protein Data Analysis

The fractions of each sample were loaded for high performance liquid chromatography separation using the Easy nLC nanoflow liquid chromatography instrument (Thermo Fisher Scientific, USA). Initially, a C18 Acclaim PepMap100 (100 μm × 2 cm) nanoViper reverse phase trap column (Thermo Fisher Scientific, USA) was connected to an EASY-Column C18-A2 reversed phase analytical column (10 cm long × 75 μm inner diameter) with 3 μm resin (Thermo Fisher Scientific, USA) and balanced using 95% buffer A (0.1% formic acid). Subsequently, the fraction was injected onto a chromatographic column and separated with buffer B (0.1% formic acid in 84% acetonitrile) using a linear gradient strategy. The gradient was set as follows: linear gradient of 0–35% buffer B for 50 min; linear gradient of 35–100% buffer B for 50–55 min; the last 5 min buffer B concentration was kept at 100%. The flow speed was controlled by IntelliFlow technology at 300 nL/min.

Mass spectrometry (MS) analysis was carried out on a Thermo Scientific Q Exactive mass spectrometer (Thermo Fisher Scientific, USA) which was run in the positive ion mode. MS1 spectra were measured in data-dependent acquisition mode with a scan range of 300–1800 *m/z* at a resolution of 70,000 (200 *m/z*). The automatic gain control target value was 1e6 and maximum injection time was set to 50 ms, while the dynamic exclusion duration was set to 60.0 s. After each full scan, 20 fragment patterns of MS2 spectra were gathered. With regard to MS2 spectra, peptides were fragmented by higher-energy collisional dissociation with a normalized collision energy of 30 eV, isolation window at 2 *m/z* and a resolution of 17,500 (200 *m/z*). The under fill ratio was defined as 0.1%.

### 2.4. Proteins Identification and Differential Proteins Abundance Analysis

The MS/MS spectra data were explored using Proteome Discoverer v.1.4 (Thermo Fisher Scientific, USA) and MASCOT engine v.2.2 (Matrix Science, London, UK). The protein database, which was used to identify protein was translated from the sequences of the transcriptomic data of our previous research [25]. The search parameter settings are shown in Supplementary Table S1. In any group, if protein abundance could not be quantified for at least two samples, then that protein was filtered. The protein abundance was log_2_-transformed and normalized, and the differential protein abundance was calculated with the linear models for microarray data (LIMMA) (v.3.52.1), using factorial design: ~genotype* estrous phase [26]. Our experimental design included two factors of the estrous phase (two levels: F and L) and genotype (two levels: WW and BB) with interactions. For screening significantly altered proteins, the thresholds were set as a foldchange > 1.2 or <−0.83, and with a *p* value <0.05, as described previously [27].

### 2.5. RNA-Seq Data Analysis

Hypothalamic transcriptomic data of three BBF, three WWF, three BBL and three WWL from our previous study [25] were obtained from the National Center for Biotechnology Information Sequence Read Archive (PRJNA672275). These ewes were the same as those that we used for the proteomics assay. The downloaded data were aligned and assembled to the sheep reference genome (oar4.0: GCF_000298735.2) by HISAT2 (v.2.0.5) and StringTie (v.1.3.2d) following the pipeline established in our previous study [28]. The read counts were calculated using the HTSeq (v.0.6.1) [29]. The DESeq2 (v.1.28.1) [30] was used to determine normalized counts and the factorial design model was used to identify differentially expressed genes which is consistent with the model used for the identification of differential abundance proteins.

### 2.6. Transcriptomic and Proteomic Data Integration

Data obtained by transcriptomic and proteomic methods were integrated by the Data Integration Analysis for Biomarker discovery using Latent variable approaches for Omics studies (DIABLO) framework of the mixOmics (v.6.20.0) to determine the correlation of features between these methodologically and biologically distinct datasets. We also searched for new biological processes that could not be found in a single dataset [31]. This method is an extension of the prediction of potential structure (also called partial least-squares) method. The mRNA count data were normalized to log_2_ (counts per million) (logCPM) using edgeR (v.3.38.1) [32]. The transcripts with logCPM < 0 in more than 75% of samples were then filtered out. The proteomic data is the filtered data mentioned above for differential protein abundance analysis. In the design matrix, the block linkage was set to 0.1, and it contained estrous stage and genotype information. The optimum number of components and variables included within the final model were determined using the “perf” and the ‘tune.block.splsda’ functions with cross-validation (3 ×  5-fold). The discriminative accuracy of the model was assessed by the area under receiver operating characteristic curves. The performance of the model was measured by the balanced error rate and overall misclassification error rate.

### 2.7. Functional Annotation and Enrichment Analysis of Biomarkers

To explore the potential role of proteins in the luteal–follicular phase transition and the role of these biomarkers in *FecB* mutations affecting fertility, Gene Ontology (GO) categories [33] and Kyoto Encyclopedia of Genes and Genomes (KEGG) [34] over-representation analyses were ran by clusterProfiler (v.4.4.4) [35]. The parameter of pAdjustMethod was the false discovery rate (FDR). The top enriched KEGG pathways and GO terms were visualized using the two clusterProfiler package functions dotplot and cnetplot.

### 2.8. Quantitative Analysis of Selected Proteins with PRM

To validate the protein abundance obtained by the TMT quantification method, the abundance of randomly selected proteins in the hypothalamus were quantified using PRM analysis (Shanghai Applied Protein Technology Co., Ltd, Shanghai, China) [36]. Tryptic peptides were prepared by following the protocol as described in the TMT analysis. We then added 20 fmol of stable isotope peptides (SAAGAFGPELSR) as calibration peptides (Thermo Fisher Scientific, USA) to 1 ug sample peptides as an internal standard reference. Before reversed-phase chromatography on an Easy nLC-1200 instrument (Thermo Fisher Scientific, USA), these peptide mixtures were desalted by homemade C18 stage tips (75 μm × 200 mm inner diameter, 3 μm resin). The liquid phase solution A and solution B were the same as those in the TMT protocol described above. The 1-h gradient of liquid chromatography was as below: 0–2 min: the solution B concentration ranged from 5% to 10% for 2–45 min, the solution B concentration ranged from 10% to 30% for 45–55 min, the solution B concentration ranged from 30% to 100% for 55–60 min, and solution B was maintained at 100%. The PRM analysis was performed using a Q Exactive HF mass spectrometer (Thermo Fisher Scientific, USA). The full MS1 scan and PRM scans were performed using the parameters described in the previous PRM analysis in our lab [27]. The peptide peak areas of each peptide were extracted by Skyline v.3.5.0 [37]. The relative peak areas of each sample were integrated and then normalized with the peak areas of the calibration peptides.

## 3. Results

### 3.1. Data Quality Control

We randomly divided 12 hypothalamus samples into three experimental groups (*n* = 4 samples in each experiment). We ensured that each group contained one of BBF, one of BBL, one of WWF and one of WWL, making them suitable for the six-plex TMT isobaric labeling strategy. The three proteomic TMT six-plex experiments identified 4938, 4974, and 4824 peptides, to a depth of 5699 unique proteins across all samples, which were annotated with Oar 4.0 (Supplementary Figure S1a). A list of the hypothalamus identified proteins with the protein sequence coverage, molecular weights and unique peptides is provided in Supplementary Table S2. The analysis of protein molecular weight distribution showed that most of the protein molecular weights were between 10 and 200 kD (Supplementary Figure S1b). Approximately 26% of the protein sequence coverage was <5% (Supplementary Figure S1c). More than 72% of the determined proteins included more than one unique peptide. Among them, the protein XP_004018043.1 contained the largest number of unique peptides, (*n* = 212) (Supplementary Table S2).

### 3.2. Data Validation

**Figure 2:**
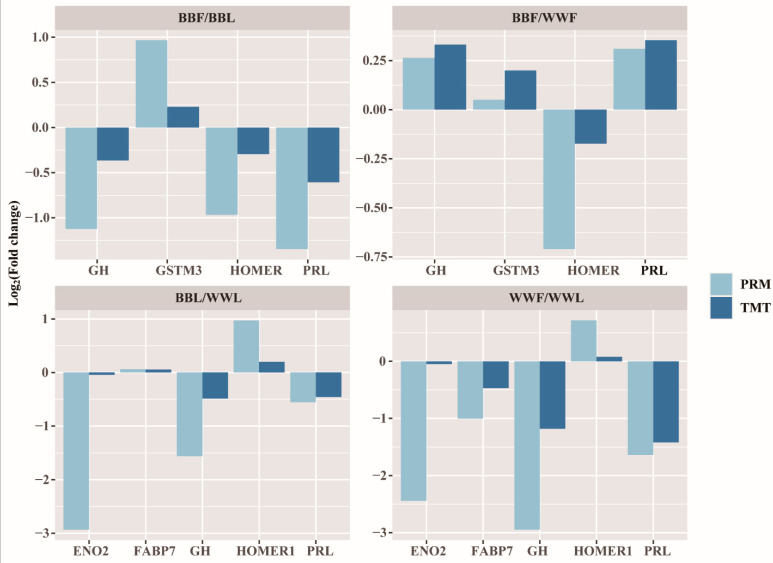
Comparison of protein abundance profiles obtained by PRM and TMT protein quantification techniques. Growth hormone (GH), glutathione S-transferase mu 3 (GSTM3), homer scaffold protein 1 (HOMER1) and prolactin (PRL) proteins were selected for protein abundance validation in the follicular phase versus the luteal phase of BB ewes (BBF/BBL groups) and in BB ewes compared with WW ewes at the follicular phase (BBF/WWF groups). PRL, GH, HOMER1, fatty acid binding protein 7 (FABP7) and enolase 2 (ENO2) proteins were selected for protein abundance validation in the follicular phase versus the luteal phase of WW ewes (WWF/WWL groups) and in BB sheep compared with WW sheep at the luteal phase (BBL/WWL groups). The log_2_fold change values were calculated between the different groups. The protein abundance measured by the PRM method compared with the TMT method for the selected genes in all four groups showed the same trend.

On the basis of the abundance of proteins in different groups in the TMT analysis, we randomly selected some important genes to validate the TMT results using the PRM method. In the BBF vs BBL and BBF vs WWF groups, prolactin (PRL), growth hormone (GH), homer scaffold protein 1(HOMER1) and glutathione S-transferase mu 3 (GSTM3) were selected for the analysis and the log_2_Fold change (log_2_FC) values were calculated in the WWF vs WWL and BBL vs WWL groups, PRL, GH, HOMER1, fatty acid binding protein 7 (FABP7) and enolase 2 (ENO2) were selected for the analysis, and the log_2_FC values were calculated. We found that the PRM results of these selected genes showed the same trends in directional abundance (up and down) as the TMT results in the different groups (Figure 2 and Supplementary Table S3).

### 3.3. Global Differential Protein Abundance

**Figure 3:**
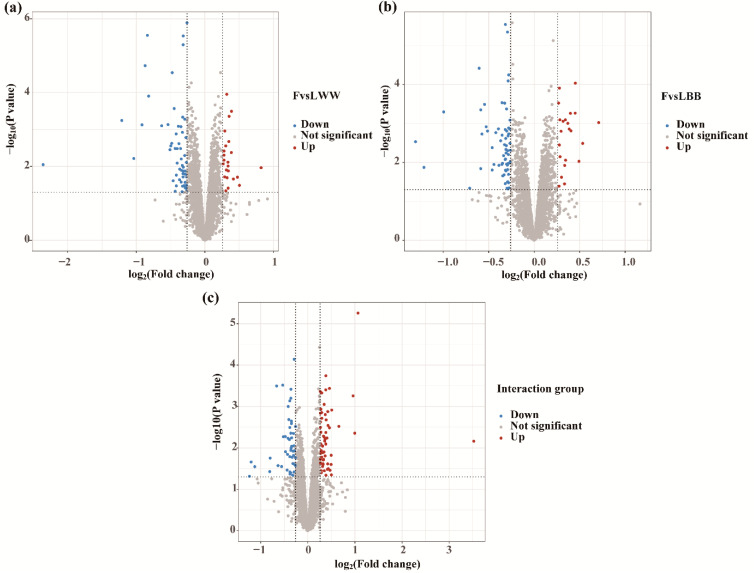
Volcano plots of DAPs. Using a foldchange >1.2 or <0.83, *p* value < 0.05 as the threshold, the upregulated and downregulated DAPs are shown separately in the FvsLWW group (**a**), the FvsLBB group (**b**) and the interaction group (**c**). FvsLWW: follicular phase relative to the luteal phase in sheep with WW genotype; FvsLBB: follicular phase relative to the luteal phase in BB sheep; Interaction: which proteins respond differently in BB compared to WW sheep.

To identify differential protein abundance, the data were normalized and analyzed using LIMMA. A generalized linear model was applied to evaluate the effects of factors (genotype and estrous phase) and their interactions using the limFit and eBayes functions. We then extracted the comparisons of interest as contrasts: which proteins change in the F relative to L in WW sheep (FvsLWW), which proteins change in the F relative to L in BB sheep (FvsLBB), and which proteins respond differently in BB compared to WW sheep (Interaction). Using a foldchange >1.2 or <0.83, and *p* value <0.05 as the threshold, 81 differentially abundant proteins (DAPs) were detected in the FvsLWW group. Among them 23 proteins were upregulated and 58 proteins were downregulated in the follicular phase (Figure 3a and Supplementary Table S4). In the FvsLBB group, 87 DAPs (22 were upregulated and 65 were downregulated) (Figure 3b and Supplementary Table S5) were screened in the follicular phase. In the interaction group, 106 DAPs (58 were upregulated and 48 were downregulated in the BB genotype) were detected (Figure 3c and Supplementary Table S6).

### 3.4. Focus on DAPs at the Luteal–Follicular Phase Transition

**Figure 4:**
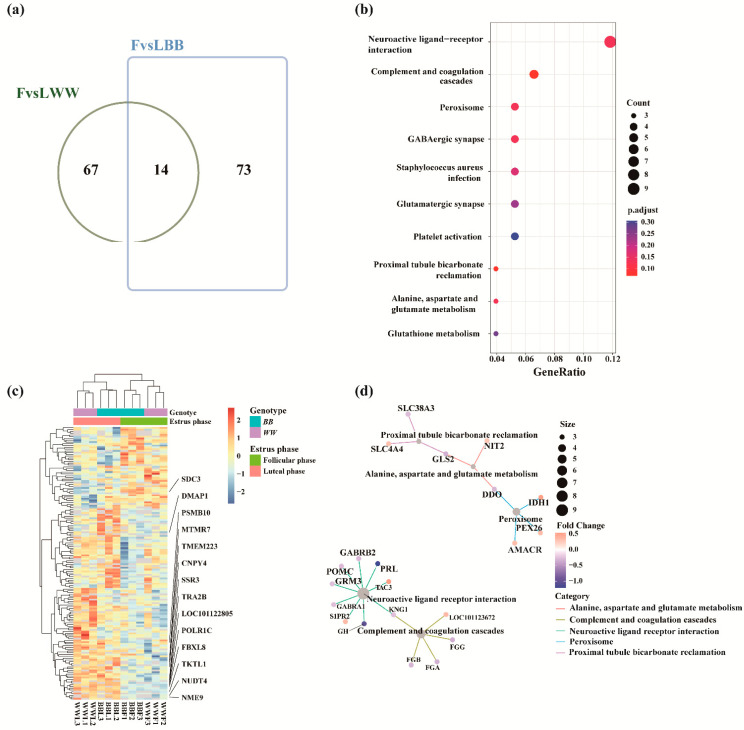
Analysis of DAPs. (**a**) Venn plot displaying the overlap of DAPs between the FvsLWW and FvsLBB groups. (**b**) Dotplot of the top 10 KEGG enrichment terms for DAPs concatenations in the FvsLWW and FvsLBB groups. pAdjust: false discovery rate (FDR). (**c**) Hierarchical cluster of DAPs in the FvsLWW and FvsLBB groups in which all of the abundance values of proteins were normalized and scaled. (**d**) Networks of the top 10 KEGG enrichment terms were visualized by cnetplot. FvsLWW: follicular phase relative to the luteal phase in WW sheep; FvsLBB: follicular phase relative to the luteal phase in BB sheep.

We focused on protein abundance differences in the luteal–follicular phase transition that may be participating in the selection and maturation of the dominant follicles. The overlaps between the FvsLWW and FvsLBB groups were visualized using an online tool (http://jvenn.toulouse.inra.fr/app/example.html (accessed on 25 October 2022)) [38]. The intersection of FvsLWW and FvsLBB groups had 14 DAPs (Figure 4a). A total abundance of 154 DAPs identified in these two groups were then subjected to hierarchical clustering heat map analysis, and the 14 DAPs in both groups showed the same trend. Except for SDC3 protein, all the proteins were down-regulated during the follicular phase (Figure 4c). We annotated all DAPs in these two groups and enriched them with GO and KEGG enrichment (Supplementary Tables S7 and S8). The networks of the top 10 KEGG enrichment terms were visualized by cnetplot (Figure 4b). Pathways of neuroactive ligand–receptor interaction and complement and coagulation cascades were linked together, and this linkage contained 13 proteins, involving PRL, GH, GABA receptors of gamma-aminobutyric acid type A receptor subunit alpha1 (GABRA1) and gamma-aminobutyric acid type A receptor subunit beta2 (GABRB2). PRL and GH are members of a common ancestral gene. They share common structural, functional and binding properties and have a wide range of biological functions [39]. The cnetplot also depicted linkages of three KEGG enriched terms (proximal tubule bicarbonate reclamation, alanine, aspartate and glutamate metabolism, peroxisome) and eight proteins involved in these terms to generate a network (Figure 4d), which were related to hypothalamic function and GnRH secretion.

### 3.5. Integrated Analysis of the Transcriptome and Proteome Screening of Potential Biomarkers Involved in STH Sheep Fertility

To identify highly correlated multi-omics (proteome and transcriptome) fertility-discriminating signatures between the WW and BB genotypes, the DIABLO framework was used to integrate mRNAs and proteins from matched samples (extracted from the same sheep). This supervised learning approach was enabled to filter optimal classification feature variables in mixed data.

**Figure 5:**
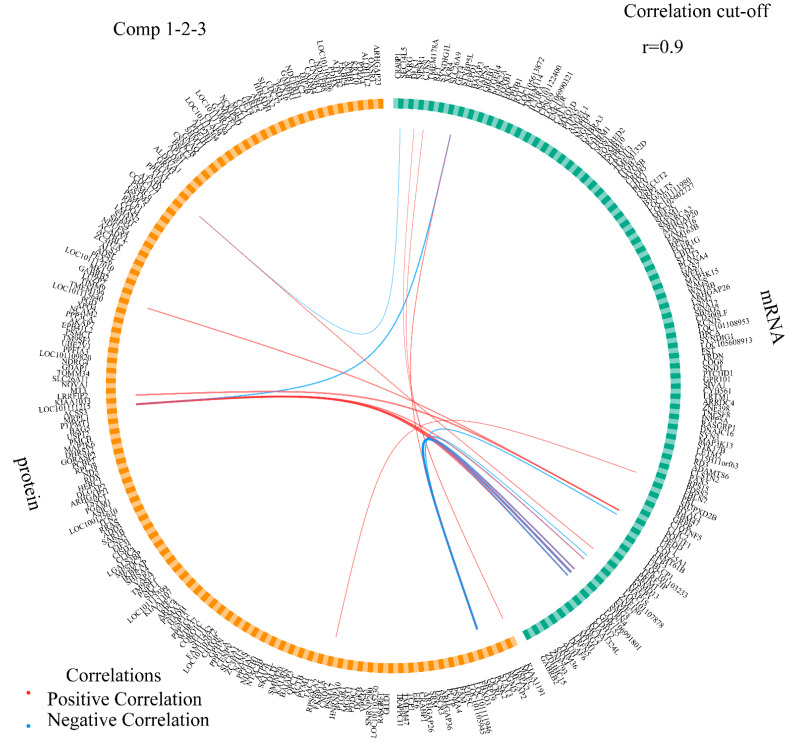
A Circos plot displays the positive (red lines) and negative (blue lines) correlations (r > 0.9) between feature variables in the quadrants.

When samples of the transcriptome and proteome were plotted individually (Figure S2a), there was no overlap among the four groups (BB and WW genotypes in the follicular and luteal phases). Similarly, no overlap was observed in a diagnostic scatterplot (plotDiablo) (Figure S2b). Proteomic and transcriptomic datasets were highly correlated, with a Pearson’s correlation score of 0.99 on two components (Figure S2b). Additionally, a scatterplot showed that the four groups were distinguishable from each other by the biomarkers obtained from the integration of the two omics datasets. The overall error rate and balanced error rate results obtained from cross-validation (3 × 5-fold) indicated that the sPLS-DA model achieved the best performance when ncomp = 3 (Figure S2c). The positive or negative correlation (Supplementary Table S10) between the abundance of protein and mRNA expression were visualized using a Circos plot (Figure 5) with ncomp = 1–3 and a correlation cutoff is 0.9. Nineteen pairs of protein-mRNA interactions were screened, of which eight pairs were negatively correlated, and 11 pairs were positively correlated. PCSK2 protein abundance was strongly correlated with the expression of seven mRNAs (*DEDD*, *LOC101107878*, *LOC106991801*, *MAP1S*, *SMG5*, *STRN4*, *STXBP5L*) and all of them were negatively correlated except for the positive correlation exhibited with the *STXBP5L* gene.

**Figure 6:**
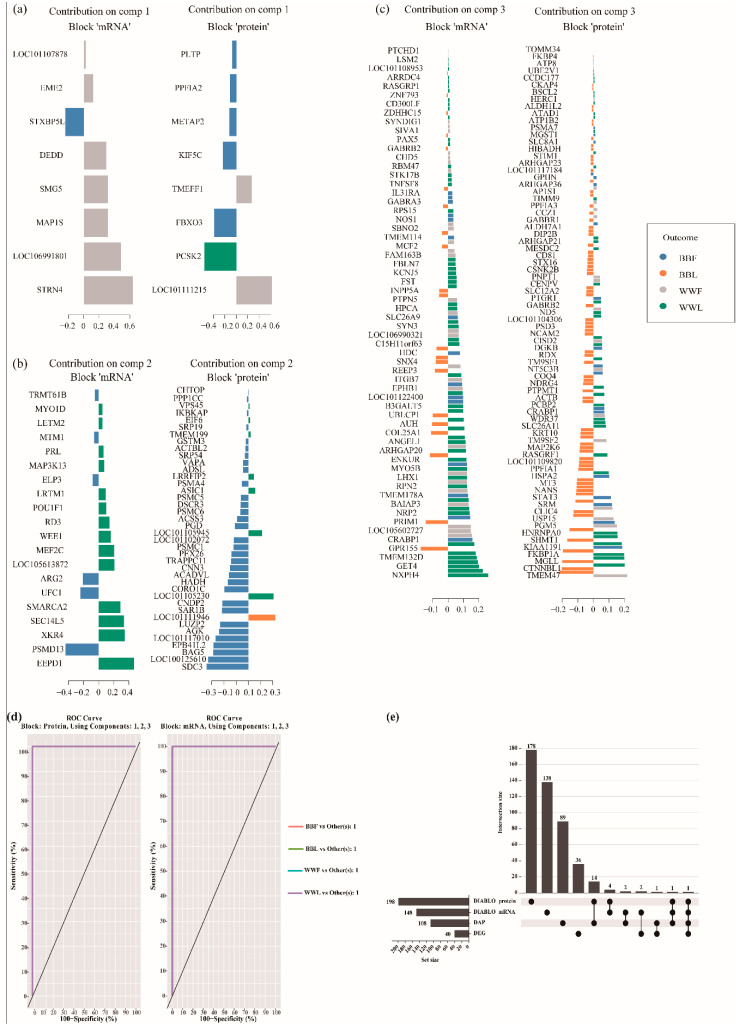
Targeted protein and mRNA panel and classification performance. DIABLO predicted the importance of multi-omics biomarkers, which were calculated on the basis of the absolute value of the loading vector weights shown for each block (protein and mRNA) and each component (component 1 (**a**), 2 (**b**), 3 (**c**)). Each biomarker is color-coded according to its association with genotype and estrous stage. (**d**) Receiver operating characteristic area under the curve (ROC AUC) provides results of 1.00 separately using mRNAs and proteins from the three components in the classifier model. (**e**) Overlap between biomarker panels identified using DIABLO and traditional single-omic multivariate approaches. This upset plot was plotted using the online tool ChiPlot (https://www.chiplot.online/ (accessed on 1 November 2022)). BBF: sheep with BB genotype in the follicular phase, BBL: sheep with BB genotype in the luteal phase, WWF: sheep with WW genotype in the follicular phase, WWL: sheep with WW genotype in the luteal phase.

The plotLoadings function was visualized by the loading weights of each feature variable in three components of two omics datasets (Figure 6a–d and Supplementary Table S9). The first component provided eight proteins and transcripts, the second component provided 40 proteins and 20 transcripts and the third component provided 150 proteins and 120 transcripts. Within each component, the feature variable at the bottom provides the greatest contribution to classification accuracy (Figure 6a–c). To further examine the genes in the transcriptome that respond differently in BB compared to WW sheep (Interaction group) 40 differentially expressed genes (DEGs) were selected from the transcriptomic data (fold change (FC) > 2 and *p* value < 0.05, padj < 0.05) (Supplementary Table S11). Then, the DAPs and DEGs screened in the interaction group were compared with discriminating variables (DIABLO_protein, DIABLO_mRNA) selected by DIABLO (Figure 6e). The feature variables determined by DIABLO were very different from those determined by traditional single-omic multivariate approaches. GABRB2 was present in three datasets (DIABLO_protein, DIABLO_mRNA and DAPs), and cellular retinoic acid binding protein 1 (CRABP1) was the intersection of these four datasets. Fourteen proteins selected by DIABLO were also DAPs in the interaction group. Only two mRNAs (*NPSR1* and *TMEM132D*) selected by DIABLO were also differentially expressed in the interaction group.

**Figure 7:**
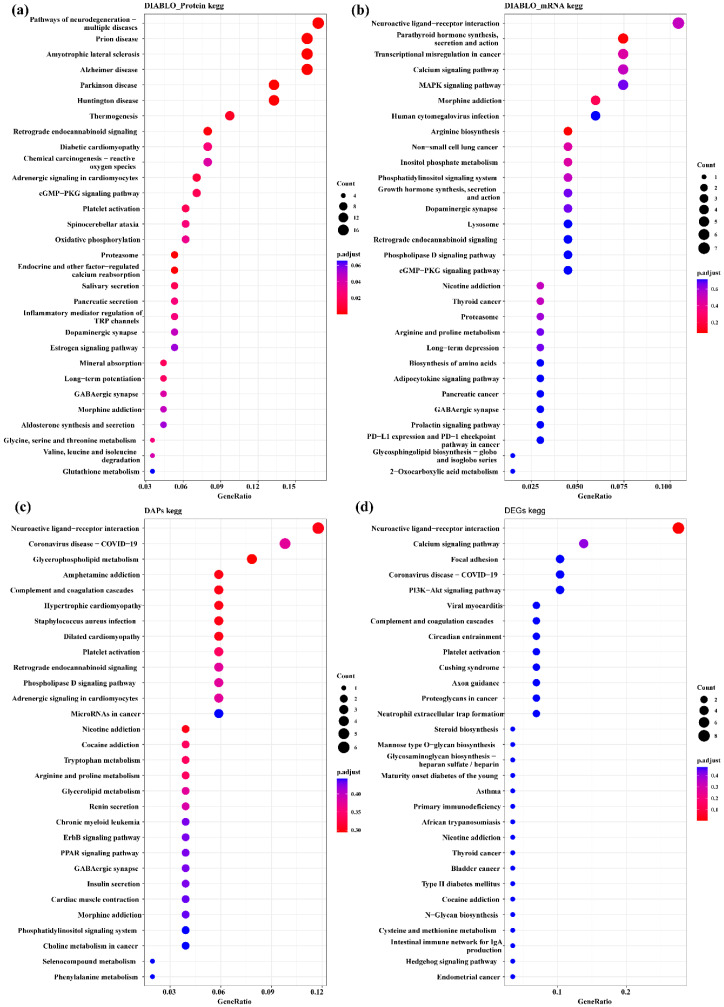
Top 30 enriched KEGG pathways of DAPs and DEGs of the interaction group and discriminating variables (DIABLO_protein, DIABLO_mRNA) selected by DIABLO. (**a**) KEGG enrichment pathways for DIABLO_protein group. (**b**) KEGG enrichment pathways for DIABLO_gene group. (**c**) KEGG enrichment pathways for DAPs of interaction group. (**d**) KEGG enrichment pathways for DEGs of interaction group.

The proteins and mRNAs from the four datasets were annotated according to KEGG enrichment analysis (Supplementary Tables S12–S15). The top 30 KEGG pathways are presented in Figure 7. It is interesting to note that some pathways were related to hypothalamic function and reproduction-related processes, such as MAPK, PI3K-Akt, the neuroactive ligand–receptor interaction, ErbB signaling pathways and GABAergic synapse. There were also several hormone-related pathways such as the prolactin signaling pathway, estrogen signaling pathway and GnRH secretion. The important biomarkers GABRB2 and PRL were enriched in the neuroactive ligand–receptor interaction pathway.

## 4. Discussion

The hypothalamus is an important regulator involved in the HPG axis and it orchestrates the complex neuroendocrine regulation of reproduction [40]. During follicular development, in *FecB* mutant sheep, GnRH accelerates preovulation follicular maturation and increases ovulation by regulating FSH and LH [11]. With the development of proteomics technology, TMT-based proteomics analysis can detect low abundance proteins with good reproducibility. Whole hypothalamic tissue of different *FecB* genotypes at different stages of follicular development were used to obtain hypothalamic protein profiles in our study. A single omic technology cannot comprehensively reveal the role of key biomarkers in the hypothalamus in the regulation of ovulation number by *FecB* mutations. To overcome this limitation, we introduced transcriptomic data. An integration analysis of proteomic and transcriptomic data was performed using the machine learning-based DIABLO framework, which reduces the complexity of the dataset generated via different platforms, and it obtained the features of important pathways.

Quantitative proteomic analysis experiments do not provide a comprehensive profile of biological regulatory networks [41]. Studies have been conducted to combine proteomic and transcriptomic data from the same sample to screen for valuable breeding genes [42,43]. In previous studies, the correlation between transcriptome and proteome data was poor owing to post-transcriptional regulation and post-translational modifications as well as experimental technical errors [44,45]. In general, mRNA splicing, changes in protein turnover and protein degradation may result in a weak correlation between the expression pattern of mRNA and the abundance of its translated proteins in tissues [46]. In multi-omics integration, mRNA expression and protein abundance data (p data points) are usually much more than the achievable sample size (n samples). In this study, to address the p ≫ n problem, we reduced the complexity of the data by selecting a subset of variables (DIABLO) [47]. Overall, our integration of two biological data types, proteomic and transcriptomic, provided greatly enhanced, powerful biological insights.

Our multi-omics approach identified several essential pathways as central to GnRH secretion and reproduction. These pathways centered on the neuroactive ligand–receptor interaction pathway, GABAergic synapse and the estrogen signaling pathway. Transcriptomic studies on pigs [48,49], goats [50] and sheep [51] have shown that neuroactive ligand–receptor interactions play a critical role in the control of reproductive processes.

GnRH neurons coordinate their activity and display intrinsic electrical activity, which requires intracellular signaling and mechanisms. Functionally, the “GnRH pulse generator” depends on a complex relationship between neurons containing norepinephrine, dopamine, 5-hydroxytryptamine, GABA, neuropeptide Y, glutamate and alanine. Glutamate and norepinephrine stimulate the HPG axis, while GABA inhibits the reproductive axis [52]. GABRA1 and GABRB2 are important biomarkers screened by combined proteomic and transcriptomic analyses, and they are enriched in the neuroactive ligand–receptor interaction pathway and GABAergic synapse pathway. These two genes belong to the GABAa receptor subunit genes (GABRs), which are heteromeric pentameric ion channels assembled from 19 different subunit heterodimers [53]. All fast GABAergic transmission occurs through the GABAa receptor in in the central nervous system [54]. GABAa receptor knockdown in GnRH neurons appears to affect the negative feedback mechanism of estrogen in mice [55]. A considerable change in the transmission of GABA to GnRH neurons occurs during an estrous cycle. In mice, GABAa receptor expression was found to be higher in L than in F [56], which is in agreement with our results. In our research, GABAa receptor expression was higher in luteal phase type BB than in WW, which suggests that an increased sensitivity for the ligand might be facilitating the GnRH surge.

PRL and GH are both endocrine hormones that are synthesized and secreted into the systemic circulation by the pituitary gland and can affect female reproductive function [57]. In this study, PRL was an important signature factor in the transcriptome that was identified by the DIABLO method, in both proteomic and transcriptomic results. PRL was also highly expressed in the luteal phase, where it was enriched in most pathways related to hypothalamic reproductive endocrine system, such as in neuroactive ligand–receptor interactions, cytokine–cytokine receptor interactions, prolactin and PI3K-Akt signaling pathways. PRL is expressed in the hypothalamus [58,59]. Although GH is not an important biomarker, its expression in the proteome is much higher in the luteal phase than in the follicular phase, and GH mRNA expression is detected in the lateral hypothalamus of the rat brain and is not associated with pituitary GH expression. GH transcripts have also been detected in the hypothalamus and extrahypothalamus regions in the chicken brain [60]. Furthermore, GH and PRL are extrapituitary hormones that can play an autocrine/paracrine role in health and disease [59]. The identification of GH and PRL in the hypothalamus suggests that these two endocrine hormones, can play important biological functions in the hypothalamus as local growth factors in paracrine/autocrine forms in the sheep hypothalamus for regulating the estrous cycle and ovulation.

FK506-binding immunoaffin (FKBP4 and FKBP5) is enriched in the estrogen signaling pathway. *FKBP4* and *FKBP5* share a similar gene structure and are involved in the regulation of steroid hormone receptor activity [61]. High *FKBP4* expression can cause infertility [62]. FKBP5 may regulate the cellular response to progesterone through a short feedback loop [63,64]. In the present study, the *FKBP5* gene in the follicular phase was significantly more highly expressed in sheep with the BB genotype than those with the WW genotype. The reason for this finding could be due to the feedback regulation of FKBP5 through progesterone, which causes the pituitary gland to secrete more FSH. By combined proteomic and transcriptomic analysis, we identified FKBP prolyl isomerase 1A (FKBP1A) as an important feature factor in the proteome, and it belongs to the FKBPs (FK506 binding-protein). FKBP1A is a common inhibitor of type I receptors of the TGF-β family [64]. FKBP1A is an important switch for TGF-β pathway activation. BMPR1B binding to ligands or non-covalent binding of molecules such as FK506 to FKBP1A can dissociate the inhibitory protein FKBP1A from BMPR1B. Subsequently, the phosphorylatable site in the GS region of BMPR1B is exposed [65]. This site is activated and phosphorylated [66], and then passes the phosphate group to the substrate R-Smad to eventually activate the TGF-β pathway [67]. The sheep *FecB* mutation (residue Q249R) is localized at the C-terminal end of the αC helix of the GS structural domain of the BMPR1B protein, which is linked to FKBP1A. This mutation enhances the binding capacity of FKBP1A and BMPR1B thus reducing TGF-β pathway activity [2]. Our study showed that FKBP1A protein abundance was lower in the BB sheep than in the WW sheep. However, there was no difference in BMPR1B protein abundance between the two genotypes; therefore, we speculate that BB sheep maintain TGF-β pathway activity by reducing FKBP1A protein abundance.

## 5. Conclusions

This hypothalamic proteomic analysis shows the abundance of protein profiles in the hypothalamus at different estrous stages with the *FecB* mutation. Extrapituitary hormones (GH and PRL) were found to be present in the hypothalamus. A combination of proteomic and transcriptomic datasets using DIABLO enabled efficient screening of important fertility biomarkers in the hypothalamus. The GABAa receptor may play a key role regulating the GnRH surge. We suggest that BB sheep maintain TGF-β pathway activity by reducing FKBP1A protein abundance. The findings of our multi-omics analysis in the hypothalamus provide fresh insights into the mechanisms by which the *FecB* mutation alters fertility through endocrine regulation. Our results also provide candidate biomarkers for sheep breeding related to reproductive endocrinology.

## Data Availability

Hypothalamic RNA-Seq data were downloaded from the National Center for Biotechnology Information Sequence Read Archive (PRJNA672275). The proteome of hypothalamus data presented in this study are available on request from the corresponding author.

## Supplementary Materials

The following supporting information can be downloaded at: https://www.mdpi.com/article/10.3390/biology12010072/s1, Figure S1: Distributions of molecular weight, Protein sequence coverage, and number of unique peptides of proteins identified in the hypothalamus; Figure S2: Illustration of N-integration supervised analysis with DIABLO. Table S1: Search parameters used for protein identification; Table S2: A list of the identified proteins in hypothalamus; Table S3: The quantification results of PRM assay of selected proteins in different groups; Table S4: The results of differential proteins abundance in FvsLWW group; Table S5: The results of differential proteins abundance in FvsLBB group; Table S6: The results of differential proteins abundance in Interaction group; Table S7: DAPs KEGG enrichment analysis in the FvsLWW and FvsLBB groups; Table S8: Enriched GO terms of DAPs in FvsLWW and FvsLBB groups; Table S9: The biomarkers selected by DIABLO framework in the proteome and transcriptome data; Table S10: The matrix of the corrlation between the abundance of protein and the expression of mRNA; Table S11: The results of differentially expressed mRNA in Interaction group; Table S12: KEGG enrichment analysis in the DAPs of interaction group; Table S13: KEGG enrichment analysis in the DEGs of interaction group; Table S14: KEGG enrichment analysis in the proteins selected by DIABLO; Table S15. KEGG enrichment analysis in the mRNAs selected by DIABLO.
