# Supplementary material for: Integrated Proteotranscriptomics of the Hypothalamus Reveals Altered Regulation Associated with the FecB Mutation in the BMPR1B Gene That Affects Prolificacy in Small Tail Han Sheep
Source: Biology (Basel). 2022 Dec 30;12(1):72. doi: 10.3390/biology12010072 (PMC9856028; doi:10.3390/biology12010072)
Supplement: Supplementary file 1 [file biology-12-00072-s001.zip › Supplementary files/supplimentary Figure S1 and S2.docx]

Supplementary Figure S1

Figure S1. Distributions of molecular weight, Protein sequence coverage, and number of unique peptides of proteins identified in the hypothalamus. (a) Distribution of protein molecular weights (kDa). (b) Distribution of [protein sequence](https://www.sciencedirect.com/topics/biochemistry-genetics-and-molecular-biology/peptide-sequence) coverage (%). (c) Venn diagram displaying the overlap of protein identification by proteomic TMT 6-plex experiments.

Supplementary Figure S2


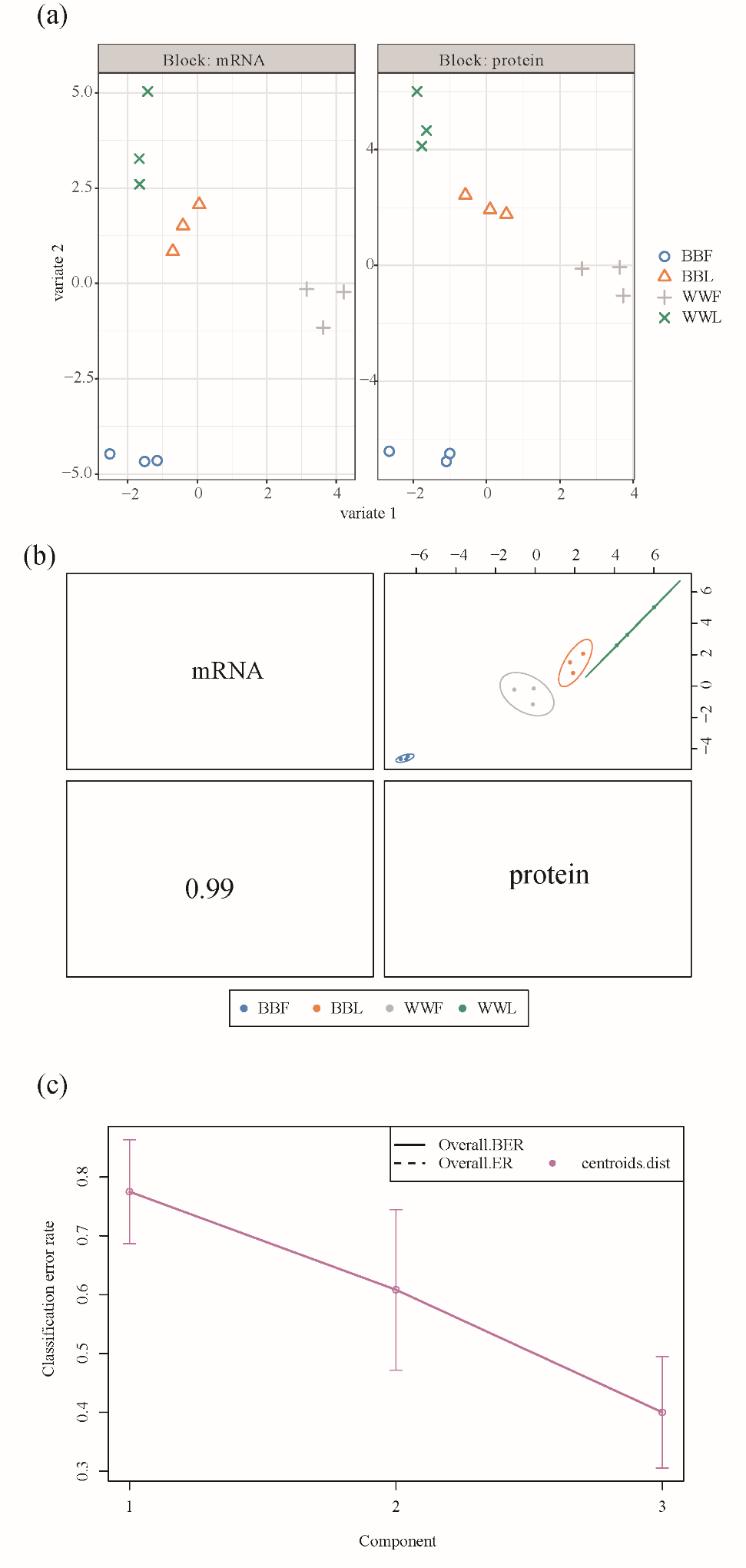


Figure S2. Illustration of N-integration supervised analysis with DIABLO. (a) Sample plot per data set highlighting the integrated protein and mRNA targets. (b) Diagnostic scatterplot displaying components 1-2 of each dataset (protein and mRNA), (c) Classification performance per component (overall and Balanced Error Rate) for prediction distances (centroids dist) using repeated stratified cross-validation (3 x 5 fold).
