# Supplementary figures and images for: Integrated Proteotranscriptomics of the Hypothalamus Reveals Altered Regulation Associated with the FecB Mutation in the BMPR1B Gene That Affects Prolificacy in Small Tail Han Sheep
Source: Biology (Basel). 2022 Dec 30;12(1):72. doi: 10.3390/biology12010072 (PMC9856028; doi:10.3390/biology12010072)

a

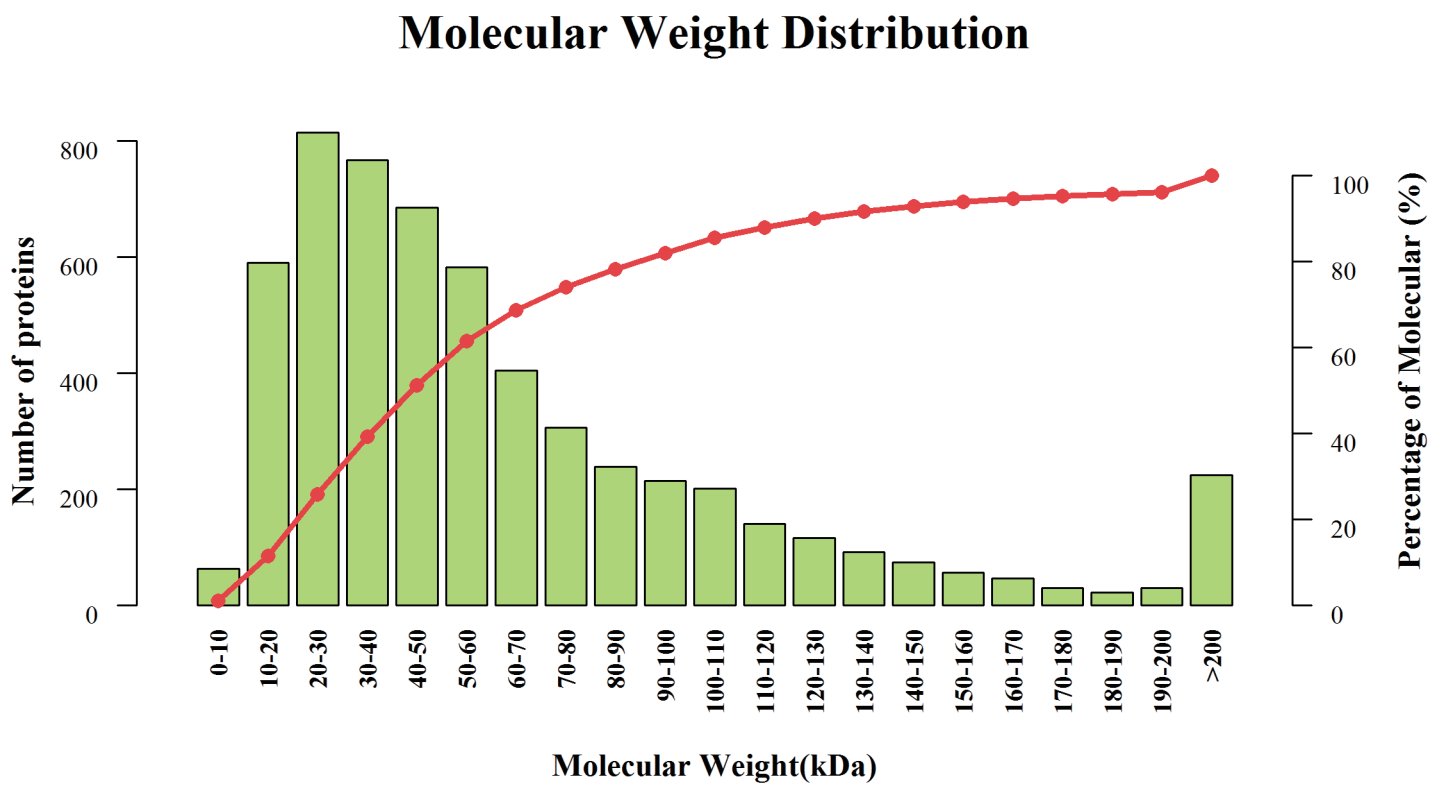

b

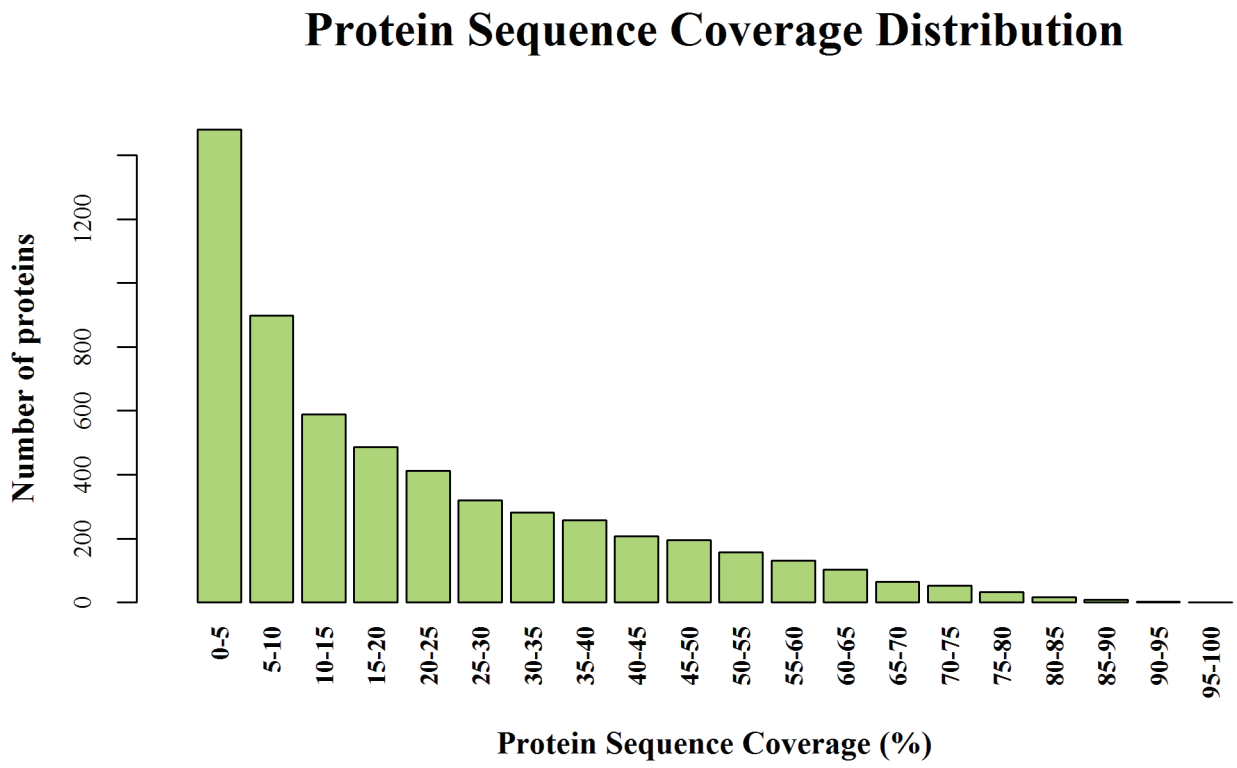

c

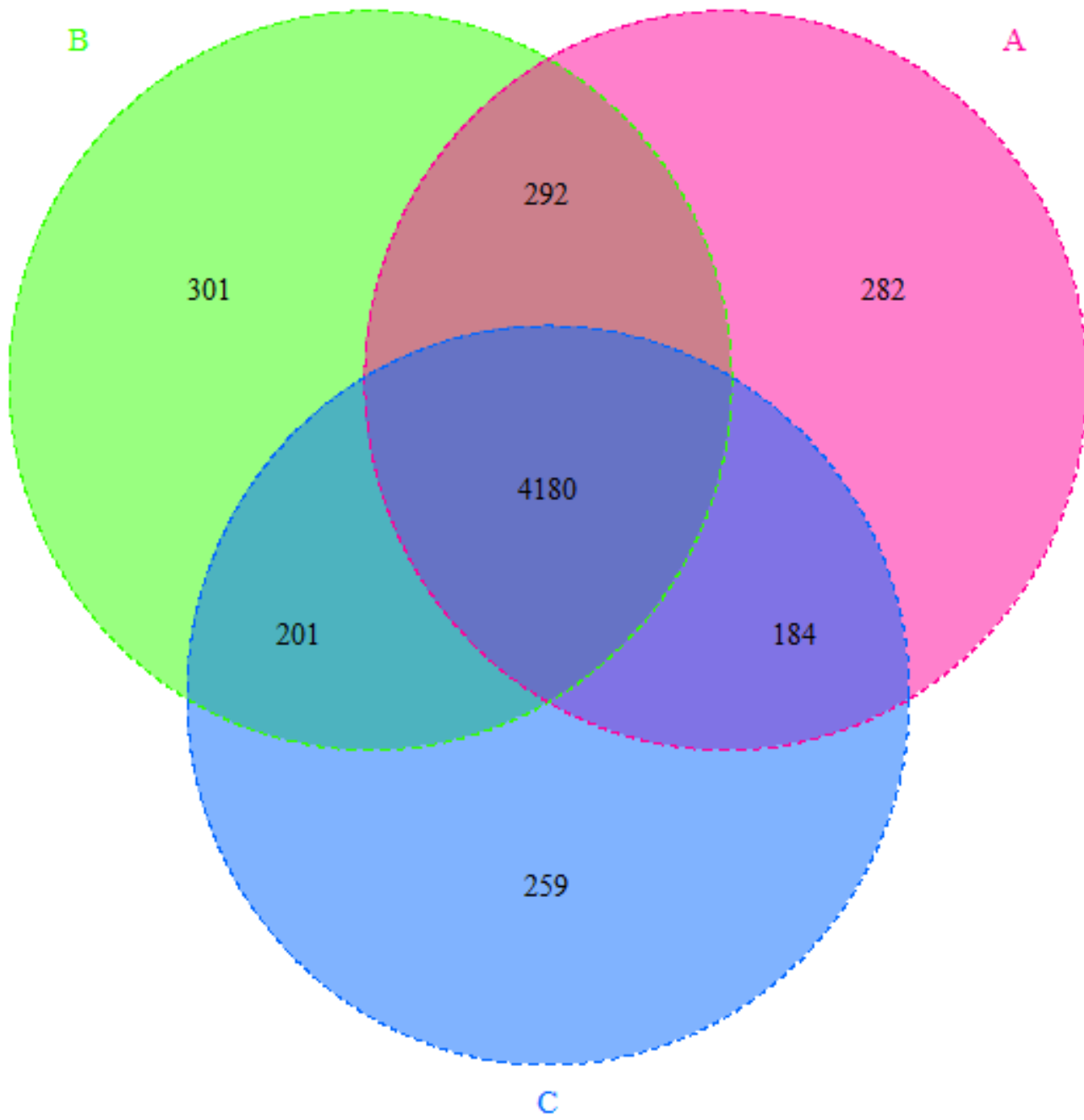

Supplement: Supplementary file 1 [file biology-12-00072-s001.zip › Supplementary files/Figure S1.pdf]

(a)

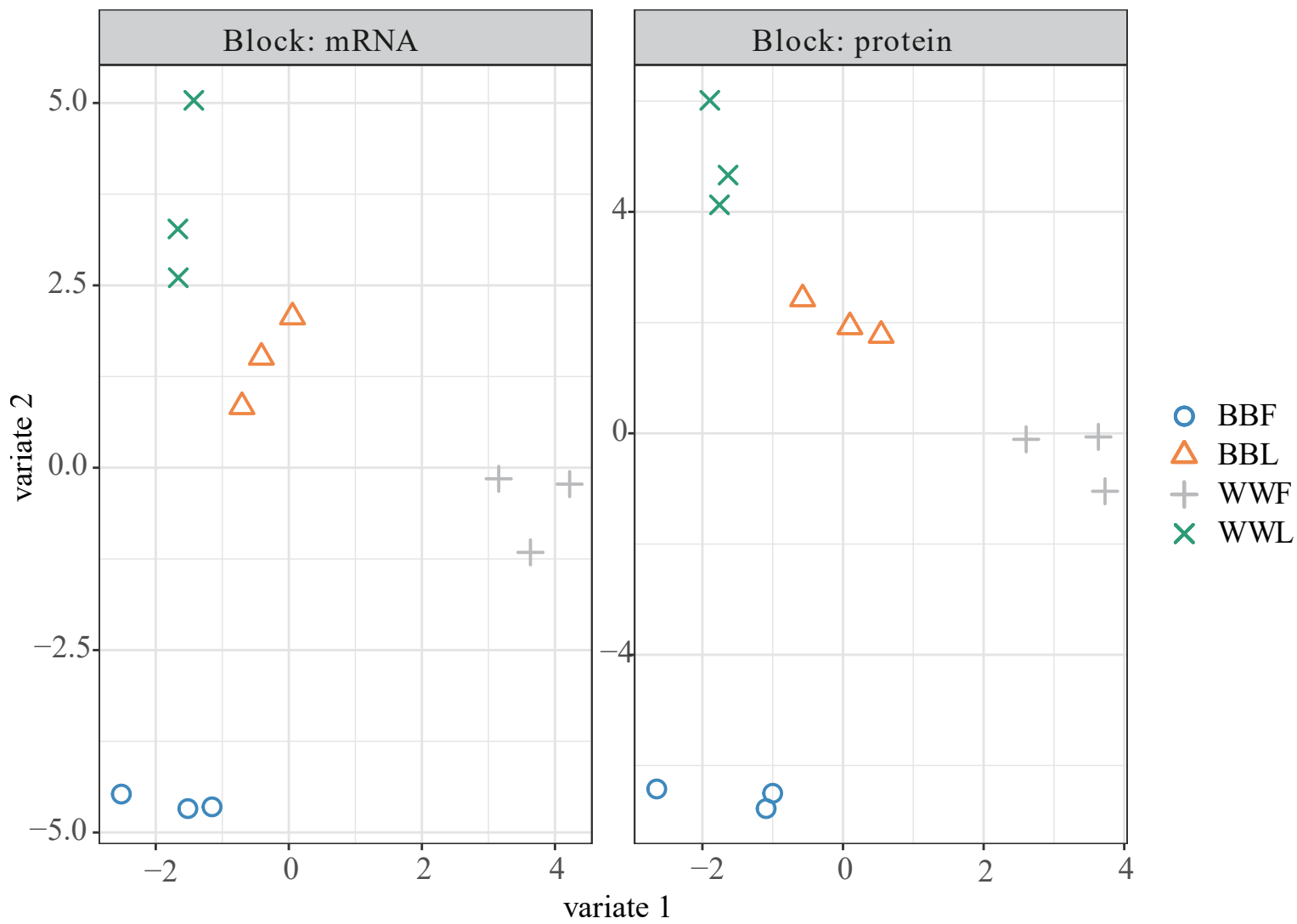

(b)

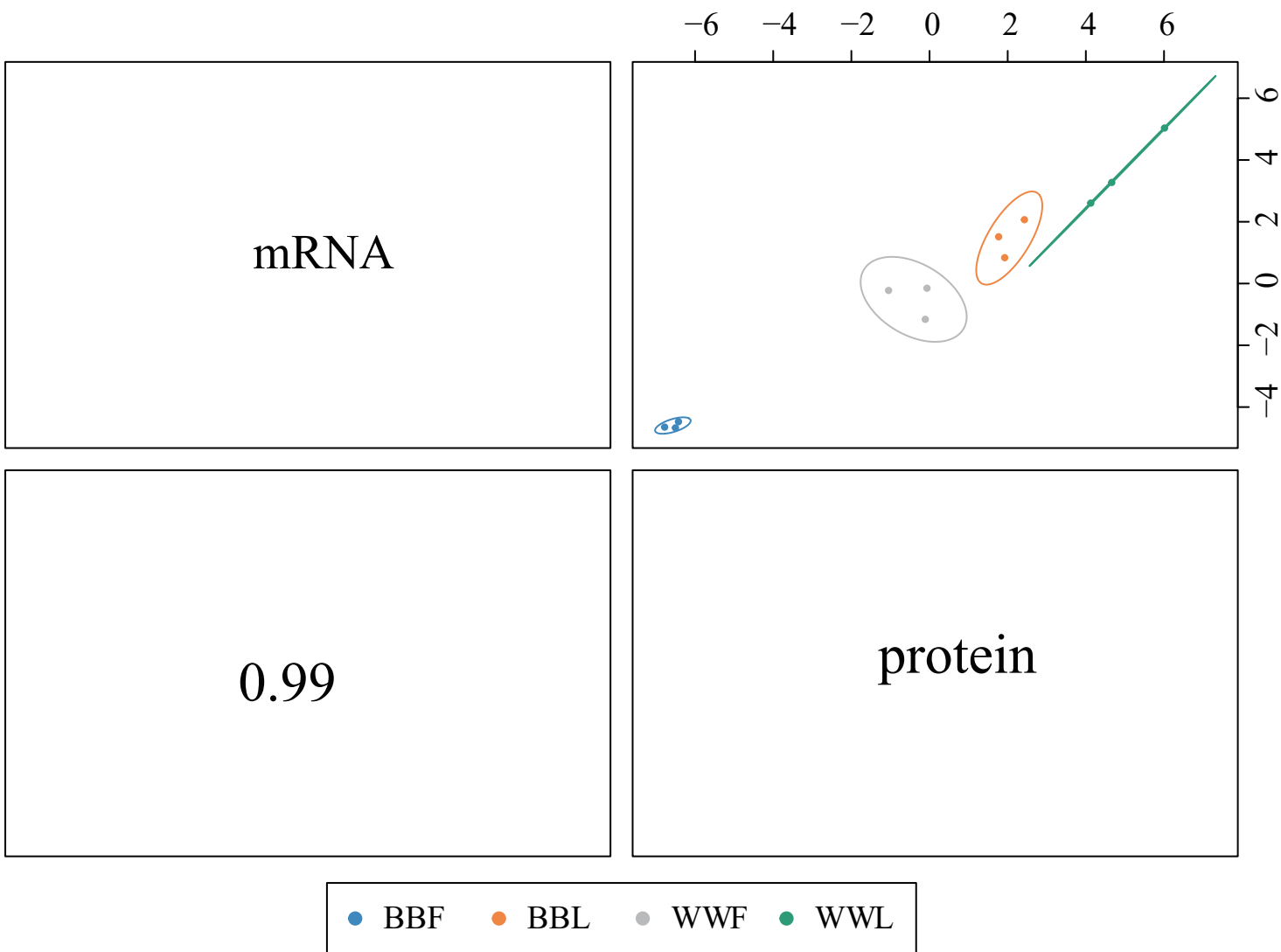

(c)

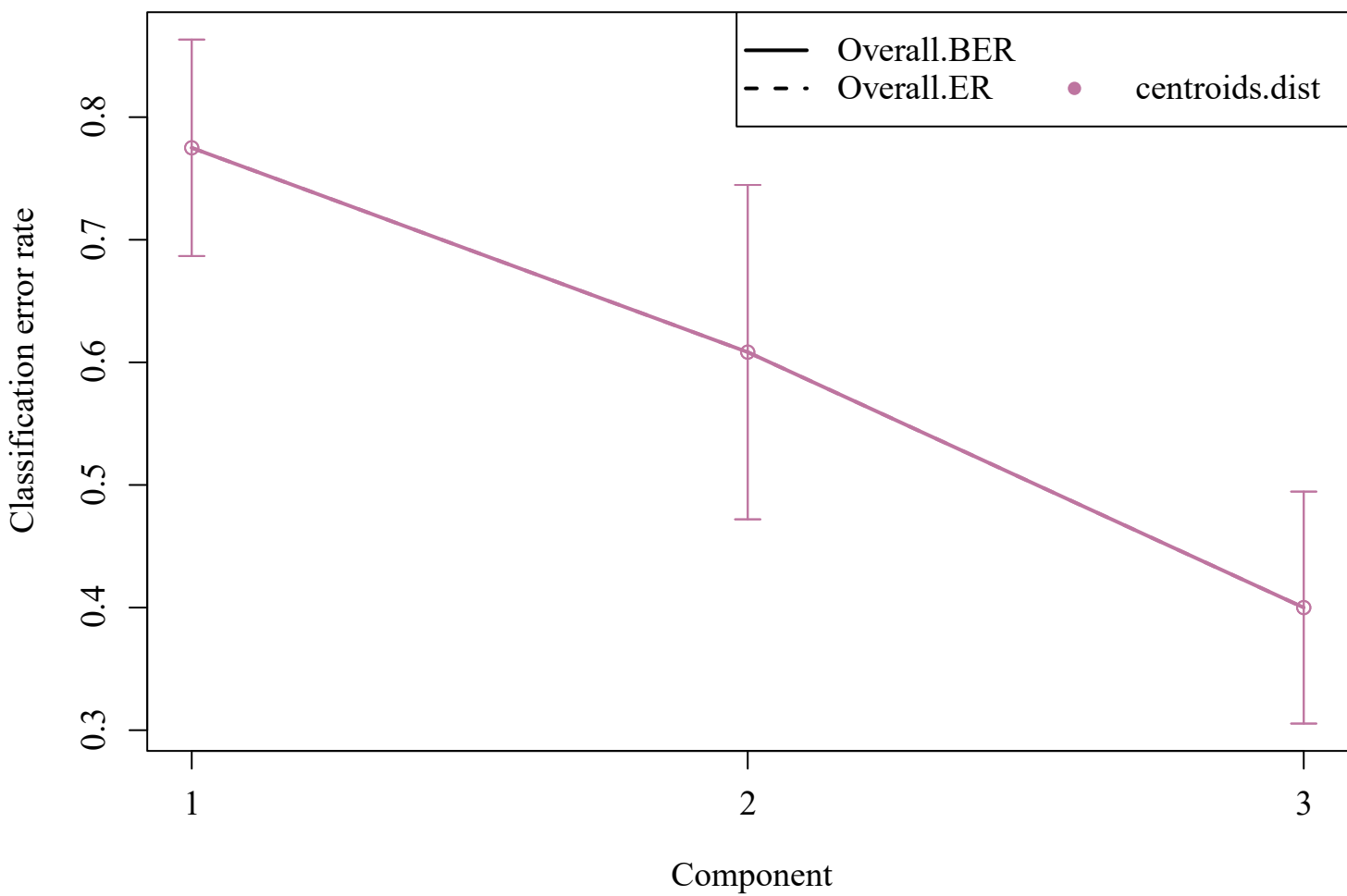

Supplement: Supplementary file 1 [file biology-12-00072-s001.zip › Supplementary files/Figure S2.pdf]
